# The biggest experience in treatment of symptomatic patients with myocardial "bridges"

**DOI:** 10.1186/1749-8090-10-S1-A155

**Published:** 2015-12-16

**Authors:** Olena Gogayeva, Gennadiy Knishov, Anatolii Rudenko, Liudmyla Dzakhoieva, Sergiy Rudenko

**Affiliations:** 1 Department of Surgical Treatment of Ischemic Heart Disease, GF "National Amosov's Institute of Cardiovascular Surgery NAMS of Ukraine", Kyiv, 03110, Ukraine

## Background/Introduction

The anomaly of location of the coronary arteries (CA) is one of the lesser known areas in the development of ischemic heart disease. Literature reports about myocardial "bridge" (MB) have usually been based on single cases and there are no algorithms of treatment patients with this anomaly. We have the largest database of the patients with MB.

## Aims/Objectives

To show our experience in treatment of symptomatic patients with MB.

## Method

During 9 years we observed 311 patients with symptomatic MB, which were diagnosed during coronary angiography. Average age of patients was 46 years. The following clinical studies have been done for all patients: ECG, echocardiography and coronary angiography.

## Results

Mostly (96,1%) MB was settled down at the LAD.

The average systolic compression was 60%. We use individual approach in treatment, which is based on the compression level for tunneled part of the CA, the presence of atherosclerotic plaque, accompanying heart pathology. 254 (81.7%) patients received medication. To standard medical therapy with B-blockers, Ca channels blockers and antiaggregants we added anxiolytics. 96.06% of patients showed a significant improvement of being. Drug-eluting stents implantation of the tunneled artery was carried out for 22 patients (7.07%), for 4 (18.1%) patients in stent restenosis appeared. 35 (11.2%) patients had surgical correction, among them 15 CA bypass grafting (CABG); 1 - supracoronary myotomy; epicardiotomy with dennervation of tunneled segment of CA had been performed for 5 patients; 2 patients with hypertrophic cardiomyopathy had undergone Morrow operation. For 12 patients with postinfarction aneurism of left ventricle we performed a CABG with resection of an aneurism of left ventricle with thrombectomy in 7 cases.

## Discussion/Conclusion

Individual approach in treating patients with MB helps to avoid life-threatening events and to improve the quality of life for patients with this anomaly. Despite the safety of endovascular procedure, indications for this minimally invasive treatment should only be performed in cases of patients who are resistant to drug therapy and have an unextended (<15 mm) tunneled segment of CA.

